# Extracting Weight of Evidence from *p*-Value via Bayesian Approach to Activation Likelihood Estimation Meta-Analysis

**DOI:** 10.3390/brainsci16010087

**Published:** 2026-01-12

**Authors:** Tommaso Costa, Jordi Manuello, Franco Cauda, Annachiara Crocetta, Donato Liloia

**Affiliations:** 1 GCS-fMRI Group, Koelliker Hospital and Department of Psychology, University of Turin, 10124 Turin, Italy; tommaso.costa@unito.it (T.C.); franco.cauda@unito.it (F.C.); annachiara.crocetta@unito.it (A.C.); donato.liloia@unito.it (D.L.); 2 FunctiOnal Neuroimaging and Complex neUral Systems (FOCUS) Laboratory, Department of Psychology, University of Turin, Via Verdi 10, 10124 Turin, Italy; 3 Neuroscience Institute of Turin, 10124 Turin, Italy; 4 Department of Social and Human Science, University of Valle D’Aosta, 11100 Aosta, Italy

**Keywords:** weight of evidence, Bayes factor, *p*-value, Activation Likelihood Estimation

## Abstract

Background: *p*-values are ubiquitous in scientific research, yet they fundamentally fail to quantify the strength of evidence for or against competing hypotheses. This limitation is particularly problematic in neuroimaging meta-analyses, where researchers need to assess how strongly the available data support specific and spatially consistent patterns of brain activation across studies. Methods: In this work, we present a practical approach that transforms *p*-values into their corresponding upper bounds on the Bayes factor, which quantify the maximum plausible evidence in favor of the alternative hypothesis given the observed data. The method is illustrated within the framework of Activation Likelihood Estimation, the most widely used coordinate-based meta-analytic technique in neuroimaging and applied to a reference dataset comprising 73 finger-tapping experiments. Results: The results show that effects traditionally classified as statistically significant using the canonical Activation Likelihood Estimation framework actually span a wide range of evidential strengths, with Bayes factor bounds varying approximately from 46 to 410. This finding reveals substantial heterogeneity in weight of evidence that is concealed by conventional threshold-based inference. Conclusion: By enabling the construction of voxel-wise maps of evidential strength while remaining fully compatible with existing analysis pipelines, the proposed approach helps to avoid common misinterpretations of *p*-values and improves the interpretability and reliability of neuroimaging meta-analytic conclusions. It therefore provides a conservative, Bayesian-inspired complement to standard significance maps.

## 1. Introduction

Null-hypothesis significance testing (NHST) and the *p*-values it produces remain the dominant inferential framework across the scientific literature. Yet *p*-values are notoriously difficult to interpret, even for experienced researchers, and their misuse has attracted increasing attention in recent years [1]. In 2016, the American Statistical Association released an influential statement outlining six principles for the responsible use of *p*-values [2], followed by further discussions and recommendations aimed at clarifying their limitations [3]. All these efforts converge on a central point: *p*-values do not answer the fundamental scientific question, namely, *how strongly do the data support one hypothesis over another?*

A common misinterpretation is to treat the *p*-value as the probability that the null hypothesis is true, for instance interpreting *p* < 0.05 as indicating a 5% chance that the null hypothesis holds and a 95% chance that the alternative hypothesis is true [4]. This interpretation is incorrect. A *p*-value instead quantifies the probability, under the null hypothesis, of observing a test statistic at least as extreme as the one obtained [5]. It therefore provides no direct assessment of evidential strength either for or against the null hypothesis.

Bayesian statistics offer a natural solution to this problem [6,7,8]. The Bayes Factor (BF) expresses how much more probable the observed data are under the alternative hypothesis than under the null, thereby providing a direct quantitative measure of evidential strength. Despite this advantage, BFs are often viewed with caution by frequentists because their computation requires specifying a prior distribution under the alternative hypothesis. However, Bayarri and colleagues [9] demonstrated that Bayes factors possess a frequentist justification for the typical problem of testing a precise null against a composite alternative, thereby bridging the conceptual gap between the two inferential paradigms.

In neuroimaging—particularly in coordinate-based meta-analyses (CBMAs)—only a few attempts have been made to transition from *p*-values to Bayesian measures of evidence [10,11,12,13]. One recent work [11] applied the minimum Bayes Factor (mBF) derived from the distribution of z-statistics under the null and alternative hypotheses, demonstrating the robustness of a Bayesian approach in both simulated and empirical CBMAs.

An alternative, complementary strategy is to convert *p*-values directly into Bayes factors, allowing researchers to reinterpret standard outputs from classical statistics in evidential terms without modifying their analysis pipelines. In the present study, we pursue this approach within the Activation Likelihood Estimation (ALE) framework [14], the most widely used CBMA technique in human neuroimaging. We make use of the method proposed by Bayarri and colleagues [9], which provides an upper bound on the Bayes factor (Bayes Factor Bound, BFB) that depends only on the observed *p*-value and does not require specifying priors. We detail this method, briefly summarize the ALE procedure, and demonstrate its application to a classical dataset of 73 finger-tapping experiments.

The paper is organized as follows. Section 2 introduces the Activation Likelihood Estimation framework and summarizes its methodological foundations. Section 3 presents the conversion of *p*-values into upper bounds on the Bayes factor and discusses their interpretation as measures of evidential strength. Section 4 illustrates the application of this approach to a reference dataset of finger-tapping experiments. Section 5 discusses methodological implications, limitations, and future directions, and Section 6 concludes.

## 2. Activation Likelihood Estimation

Activation Likelihood Estimation (ALE) is a voxel-based, data-driven, and hypothesis-free meta-analytic technique developed to quantify the spatial convergence of activation patterns across neuroimaging studies [15]. The method models each reported activation focus—specified as an x–y–z coordinate in standardized stereotaxic space—as a three-dimensional Gaussian probability distribution. This probabilistic representation reflects the spatial uncertainty associated with the true underlying location of the activation.

Formally, for a given neuroimaging study S, each reported coordinate f is associated with a modeled activation (MA) map defined as:

(1) $$P_{S,f}(v)=c\;N(v|f,\sigma^{2}I),$$

where v denotes a voxel in brain space, N(⋅) is a three-dimensional Gaussian probability density function with mean equal to the reported coordinate f and isotropic covariance σ^2^I, and c is a normalization constant ensuring that the densities across voxels sum to 1. The parameter σ controls the width of the Gaussian kernel and is empirically determined as a function of the sample size n of the original study [16]. In this formulation, P_{S,f}(v) does not represent a likelihood function in the strict statistical sense, nor a probability in the Bayesian sense, but rather a modeled activation density that quantifies the spatial uncertainty associated with the reported activation focus and expresses the relative plausibility that voxel v corresponds to its true underlying location.

After constructing an MA map for each reported focus, ALE combines them into a study-level map:

(2) $$P_{S}(v)=1-\prod_{f\in S}(1-P_{S,f}(v)),$$

which represents the probability that study S reports an activation at voxel v. This expression corresponds to the union of independent probabilistic events, assuming that individual foci within a study reflect distinct activation attempts.

At the group level, ALE aggregates the study-level maps across all included experiments to estimate the convergence of activation patterns. The distribution of ALE values expected under the null hypothesis of spatial independence is approximated via permutation testing. Specifically, random MA maps are generated under the assumption that reported coordinates arise independently across studies, and the empirical null distribution is used to derive *p*-values for each voxel [17,18].

Standard ALE implementations—such as those in the GingerALE software [16]—provide both thresholded and unthresholded ALE maps, together with the corresponding voxel-wise *p*-value and Z-score maps. This makes ALE especially suitable for reinterpretation using Bayesian evidence measures, since the transformation from *p*-values to Bayes Factor Bounds (BFBs) can be applied directly to the output without modifying the underlying meta-analytic pipeline.

It is worth noting that, although quantities such as modeled activation values are often informally referred to as probabilities in the ALE literature, they do not represent probabilities in the strict Bayesian sense, nor likelihood functions of parameters given data. Rather, they are best interpreted as modeled activation densities that quantify spatial uncertainty associated with reported coordinates and serve as intermediate constructs for estimating convergence across studies.

## 3. Converting *p*-Values to Bayes Factors

Several approaches have been proposed to approximate Bayes factors directly from *p*-values [19]. One of the most widely used formulations provides an *upper bound* on the Bayes factor in favor of the alternative hypothesis $H_{1}$ over the null hypothesis $H_{0}$. For a two-sided test, the Bayes Factor Bound (BFB) is given by:

(3) $$BFB(p)=\frac{1}{-e\;pln(p)},$$

where p is the observed *p*-value and e is the base of natural logarithms. This expression holds under broad regularity conditions and yields the maximum plausible Bayes factor consistent with the reported *p*-value.

However, ALE studies typically rely on one-sided tests, since activation likelihoods cannot assume negative values [11]. To account for this, the appropriate expression is obtained by replacing p with its one-sided equivalent. This yields:

(4) $${BFB}_{ALE}(p)=\frac{1}{-e\;\left(\frac{p}{2}\right)\ln\left(\frac{p}{2}\right)\;},$$

which ensures that the BFB correctly reflects the evidential weight for the presence of activation (i.e., the effect of interest in ALE-based meta-analyses).

Like standard Bayes factors, the BFB quantifies the evidence for $H_{1}$ relative to $H_{0}$ and ranges from 0 to +∞. Although the BFB is not a full Bayes factor—because it does not rely on specifying a prior for the alternative—it provides a conservative estimate of the maximum evidence compatible with the observed *p*-value. In this sense, it represents a principled Bayesian reinterpretation of classical statistical results.

Assuming equal prior odds, $P(H_{0})=P(H_{1})$, the BFB can be transformed into an upper bound on the posterior probability of the alternative hypothesis:

(5) $$P(H_{1}|data)\leq \frac{BFB}{1+BFB},$$

which offers an intuitive measure of how strongly the observed data support the presence of a consistent activation across studies.

The interpretation of evidential strength can follow the conventional categories proposed by Kass and Raftery [20], based on the logarithm of the Bayes factor. These categories are summarized in Table 1, which provides qualitative descriptors that can be applied directly to the BFB. These categories apply equally to the BFB because it represents a formally valid upper bound on the true Bayes factor. Using this framework, voxel-wise BFB values can be directly mapped onto qualitative descriptors such as “moderate,” “strong,” or “decisive” evidence for activation.

## 4. Application of the Bayes Factor Bound Method to the Activation Likelihood Estimation Environment

As proof of concept, we applied the proposed BFB approach to a standard ALE meta-analysis. Specifically, we analyzed a pooled dataset of 73 finger-tapping experiments from Laird and colleagues [21]. The ALE analysis was conducted using the GingerALE software package (v.3.0.2) [16], with state-of-the-art parameters [18]: family-wise error (FWE) correction at the cluster level (*p* < 0.05), a cluster-forming threshold of *p* < 0.001, and 1000 permutations to estimate the empirical null distribution.

The ALE procedure yields both a thresholded activation map and a voxel-wise *p*-value map. The latter can be directly transformed into a voxel-wise BFB map using the method described in the previous section. Operationally, for every voxel we extracted the corresponding *p*-value and applied Equation (4). The resulting BFB values were then converted into descriptive weight-of-evidence (WoE) categories using Table 1, allowing an intuitive reinterpretation of the strength of support for consistent activation across studies.

**Figure 1:**
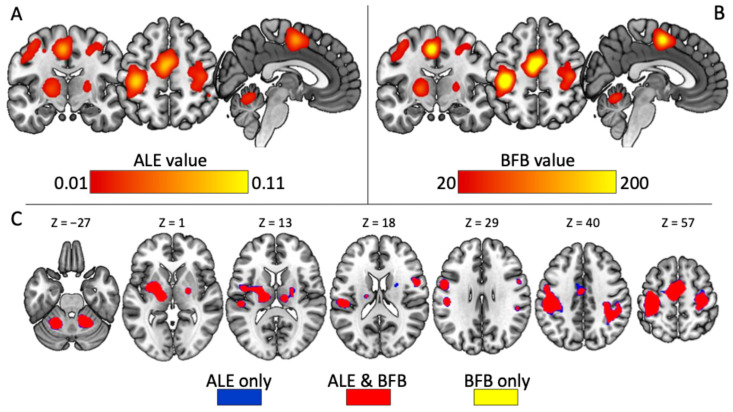
(**A**) Brain clusters showing converging activation patterns obtained with classical ALE analysis, thresholded using cluster-level FWE correction (*p* < 0.05) and a cluster-forming threshold of *p* < 0.001. (**B**) Voxel-wise Weight of Evidence (WoE) map derived from the Bayes Factor Bound (BFB), obtained by applying Equation (4) to the ALE *p*-value map. (**C**) Overlay comparison of the two approaches. Red voxels are identified by both methods; blue voxels are significant only in the classical ALE analysis; yellow voxels show substantial WoE but did not survive classical thresholding. All slices are displayed in neurological convention (left = L, right = R). ALE = Activation likelihood estimation; BFB = upper bound on the Bayes Factor.

Figure 1A shows the conventional ALE results obtained using FWE correction. Figure 1B displays the corresponding WoE map obtained from the BFB transformation, while Figure 1C provides an overlay comparison of the two approaches. Although the spatial patterns identified by the two methods overlap substantially, the BFB map reveals pronounced variability in evidential strength among voxels that are all deemed “significant” under the classical ALE framework. In other words, voxels sharing the same thresholded *p*-value can correspond to markedly different weights of evidence—sometimes differing by an order of magnitude.

Peak coordinates and their corresponding ALE values, *p*-values, and BFB-derived WoE values are reported in Table 2. These results illustrate the central message of this work: the classical significance map compresses a wide range of evidential strengths into a binary significant/non-significant dichotomy, whereas the BFB approach highlights meaningful gradations in the support for consistent activation patterns.

Overall, these findings demonstrate that a posterior probability upper-bound map and a voxel-wise WoE map can be straightforwardly derived from standard ALE outputs. The proposed approach requires no modification to existing ALE workflows and provides an interpretable, conservative, and Bayesian-inspired enhancement to traditional meta-analytic inference.

## 5. Methodological Considerations

One might argue that the maps obtained with the two approaches (Figure 1A,B) identify broadly similar patterns of brain involvement, raising the question of whether the BFB transformation provides any true additional insight. However, what changes fundamentally is not *where* activation appears, but *how strongly* the data support the presence of consistent activation across studies. Classical ALE inference relies on threshold-based NHST and therefore treats all significant voxels as equivalent. In contrast, the BFB-based WoE map reveals substantial heterogeneity in evidential strength among voxels that share the same nominal significance status. This difference is particularly important when interpreting meta-analytic results or comparing brain regions within or across studies.

A central aspect of this reinterpretation concerns the explicit formulation of hypotheses in ALE meta-analysis. Under the null hypothesis, reported foci arise randomly and independently across studies; under the alternative hypothesis, activation is consistently observed in a specific region. In the present work, we assumed equal prior odds, $P(H_{0})=P(H_{1})$. In many neuroimaging contexts—such as tasks with robust, well-established activation patterns—this assumption may in fact underestimate the true prior probability of consistent activation [22]. Consequently, the WoE values presented here are likely conservative rather than inflated, reflecting the maximum plausible evidence compatible with the observed *p*-values.

It is important to emphasize the limitations of the BFB. Like *p*-values, BFBs provide evidence *against* the null hypothesis but not affirmative evidence *for* it. They cannot quantify support for $H_{0}$, nor can they replace full Bayesian parameter estimation. In addition, different formulas exist for the conversion of *p*-values into Bayes factors. Alternative proposals—such as the calibration suggested by Held and Ott [23]—should be examined in future work to determine which transformation yields the most robust and interpretable results in the neuroimaging domain.

These considerations highlight that the added value of the BFB approach lies not in altering the ALE algorithm, but in reframing its output in evidential terms. For example, researchers deciding whether to include marginally significant foci in downstream analyses—such as network-level inference or summary statistics—may reach different conclusions when viewing a WoE map rather than a traditional thresholded statistical map. Thus, the BFB method complements existing ALE procedures by providing an interpretable, model-agnostic measure of evidential strength, thereby facilitating more nuanced and transparent meta-analytic decisions.

Future research may extend the present approach in several directions. First, alternative calibrations for converting *p*-values into Bayesian measures of evidence could be systematically compared to assess their robustness and interpretability in neuroimaging meta-analyses. Second, the proposed evidential reinterpretation could be applied to coordinate-based meta-analytic methods beyond Activation Likelihood Estimation. Finally, integrating upper-bound evidence maps with fully Bayesian meta-analytic frameworks may help to further clarify the relationship between classical significance testing and Bayesian inference in large-scale neuroimaging studies.

## 6. Conclusions

This study introduces a practical solution to a longstanding problem in neuroimaging meta-analysis: the systematic misinterpretation of statistical significance. By transforming *p*-values into Bayes Factor Bounds (BFBs), researchers can directly assess the maximum plausible weight of evidence supporting consistent activation, rather than relying solely on arbitrary significance thresholds. This evidential reinterpretation does not require any modification to existing ALE workflows and can be applied directly to standard *p*-value maps generated by tools such as GingerALE.

The resulting voxel-wise WoE maps reveal substantial variability in evidential strength that is otherwise concealed by threshold-based NHST. In doing so, they provide a more transparent and interpretable basis for meta-analytic inference, highlighting where evidence is genuinely strong and where it is merely sufficient to pass a statistical threshold. Because BFBs represent conservative upper bounds, the inferences drawn from them err on the side of caution, avoiding overstatement of evidential support.

We therefore recommend that future neuroimaging meta-analyses report both traditional ALE significance maps and complementary WoE maps derived through the BFB transformation. This dual reporting strategy preserves continuity with the existing literature while enabling a gradual shift toward evidence-based interpretation. By bridging frequentist and Bayesian perspectives, the proposed approach offers a pragmatic and accessible avenue for improving the reliability, reproducibility, and interpretability of coordinate-based meta-analytic research in human neuroimaging.

## Figures and Tables

**Table 1:** Qualitative interpretation of evidential strength according to Kass and Raftery [20]. These categories apply to both Bayes Factors (BF) and Bayes Factor Bounds (BFB).

log_10_(BF)	Evidential Strength	Interpretation
0	None	$\mathrm{No}\;\mathrm{evidence}\;\mathrm{for}\;H_{1}$
0.47	Moderate	$\mathrm{Moderate}\;\mathrm{evidence}\;\mathrm{for}\;H_{1}$
1.00	Strong	$\mathrm{Strong}\;\mathrm{evidence}\;\mathrm{for}\;H_{1}$
1.47	Very strong	$\mathrm{Very}\;\mathrm{strong}\;\mathrm{evidence}\;\mathrm{for}\;H_{1}$
2.00	Decisive	$\mathrm{Decisive}\;\mathrm{evidence}\;\mathrm{for}\;H_{1}$

**Table 2:** Peak coordinates from the ALE analysis of Laird et al. [21], with corresponding ALE values, *p*-values, and Weight of Evidence (WoE) derived from the Bayes Factor Bound (BFB). Coordinates are in Talairach space.

Cluster	x	y	z	Anatomical Label (Brodmann Area)	ALE Value	*p*-Value	WoE (BFB)
1	−40	−26	50	Left Postcentral Gyrus (BA 3)	0.11267543	5.6 × 10^−45^	410
1	−46	−40	46	Left Inferior Parietal Lobule (BA 40)	0.043866318	4.86 × 10^−06^	100
1	−54	2	28	Left Precentral Gyrus (BA 6)	0.032762058	4.93 × 10^−02^	64
1	−50	−26	18	Left Postcentral Gyrus (BA 40)	0.03167647	1.15 × 10^−01^	62
2	34	−32	46	Right Postcentral Gyrus (BA 3)	0.03909217	2.88 × 10^−04^	85
2	38	−20	54	Right Precentral Gyrus (BA 4)	0.038976684	3.16 × 10^−04^	84
2	46	−42	46	Right Inferior Parietal Lobule (BA 40)	0.034640647	1.11 × 10^−02^	70
2	24	−16	48	Right Precentral Gyrus (BA 6)	0.028492466	1.32 × 10^−01^	48
3	−4	−8	52	Left Medial Frontal Gyrus (BA 6)	0.09459096	1.48 × 10^−28^	320
4	−22	−8	4	Left Lentiform Nucleus (Lateral Globus Pallidus)	0.049706254	2.68 × 10^−09^	122
4	−32	−4	6	Left Claustrum	0.045675024	9.94 × 10^−08^	107
4	−16	−18	8	Left Thalamus (VPL)	0.043585688	6.20 × 10^−06^	102
5	16	−50	−20	Right Cerebellum (Dentate)	0.07625713	1.06 × 10^−19^	234
5	4	−58	−14	Right Cerebellum (Declive)	0.02935449	6.88 × 10^−01^	54
6	−22	−54	−24	Left Cerebellum (Culmen)	0.04886212	5.80 × 10^−08^	122
7	22	−8	4	Right Lentiform Nucleus (Lateral Globus Pallidus)	0.028590135	1.22	51
7	12	−18	12	Right Thalamus (VPL)	0.027081696	3.76 × 10^−01^	46

## Data Availability

The original data presented in the study are openly available in FigShare at https://doi.org/10.6084/m9.figshare.31016854 (accessed on 7 January 2026).

## References

[1] Siegfried T. (2010). Odds are, it’s wrong: Science fails to face the shortcomings of statistics. Sci. News.

[2] Wasserstein R.L., Lazar N.A. (2016). The ASA Statement on p-Values: Context, Process, and Purpose. Am. Stat..

[3] Wasserstein R.L., Schirm A.L., Lazar N.A. (2019). Moving to a World Beyond “p < 0.05.”. Am. Stat..

[4] Greenland S., Senn S.J., Rothman K.J., Carlin J.B., Poole C., Goodman S.N., Altman D.G. (2016). Statistical tests, P values, confidence intervals, and power: A guide to misinterpretations. Eur. J. Epidemiol..

[5] Benjamin D.J., Berger J.O., Johannesson M., Nosek B.A., Wagenmakers E.J., Berk R., Bollen K.A., Brembs B., Brown L., Camerer C. (2018). Redefine statistical significance. Nat. Hum. Behav..

[6] Liloia D., Costa T., Cauda F., Manuello J. (2024). Building diagnostic neuroimaging biomarkers for psychiatric disorders using reverse inference approaches: A viable route?. Adv. Clin. Exp. Med..

[7] Rostgaard K. (2023). Simple nested Bayesian hypothesis testing for meta-analysis, Cox, Poisson and logistic regression models. Sci. Rep..

[8] Mulder J., van Aert R.C. (2025). Bayes factor hypothesis testing in meta-analyses: Practical advantages and methodological considerations. Res. Synth. Methods.

[9] Bayarri M.J., Benjamin D.J., Berger J.O., Sellke T.M. (2016). Rejection odds and rejection ratios: A proposal for statistical practice in testing hypotheses. J. Math. Psychol..

[10] Costa T., Manuello J., Ferraro M., Liloia D., Nani A., Fox P.T., Lancaster J., Cauda F. (2021). BACON: A Tool for Reverse Inference in Brain Activation and Alteration.

[11] Costa T., Liloia D., Cauda F., Fox P.T., Mutta F.D., Duca S., Manuello J. (2023). A minimum Bayes Factor based threshold for activation likelihood estimation. Neuroinformatics.

[12] Kang J., Nichols T.E., Wager T.D., Johnson T.D. (2014). A Bayesian hierarchical spatial point process model for multi-type neuroimaging meta-analysis. Ann. Appl. Stat..

[13] Zhang L., Guindani M., Vannucci M. (2015). Bayesian models for functional magnetic resonance imaging data analysis. Wiley Interdiscip. Rev. Comput. Stat..

[14] Turkeltaub P.E., Eden G.F., Jones K.M., Zeffiro T.A. (2002). Meta-analysis of the functional neuroanatomy of single-word reading: Method and validation. Neuroimage.

[15] Costa T., Ferraro M., Manuello J., Camasio A., Nani A., Mancuso L., Cauda F., Fox P.T., Liloia D. (2024). Activation Likelihood Estimation Neuroimaging Meta-Analysis: A Powerful Tool for Emotion Research. Psychol. Res. Behav. Manag..

[16] Eickhoff S.B., Laird A.R., Grefkes C., Wang L.E., Zilles K., Fox P.T. (2009). Coordinate-based activation likelihood estimation meta-analysis of neuroimaging data: A random-effects approach based on empirical estimates of spatial uncertainty. Hum. Brain Mapp..

[17] Eickhoff S.B., Bzdok D., Laird A.R., Kurth F., Fox P.T. (2012). Activation likelihood estimation meta-analysis revisited. Neuroimage.

[18] Eickhoff S.B., Nichols T.E., Laird A.R., Hoffstaedter F., Amunts K., Fox P.T., Bzdok D., Eickhoff C.R. (2016). Behavior, sensitivity, and power of activation likelihood estimation characterized by massive empirical simulation. Neuroimage.

[19] Sellke T., Bayarri M.J., Berger J.O. (2001). Calibration of *p* values for testing precise null hypotheses. Am. Stat..

[20] Kass R.E., Raftery A.E. (1995). Bayes factors. J. Am. Stat. Assoc..

[21] Laird A.R., Robbins J.M., Li K., Price L.R., Cykowski M.D., Narayana S., Fox P.T. (2008). Modeling motor connectivity using TMS/PET and structural equation modeling. Neuroimage.

[22] Friston K.J., Glaser D.E., Henson R.N., Kiebel S., Phillips C., Ashburner J. (2002). Classical and Bayesian inference in neuroimaging: Applications. Neuroimage.

[23] Held L., Ott M. (2018). On p-values and Bayes factors. Annu. Rev. Stat. Its Appl..

